# Case of Obstetrics

**Published:** 1830

**Authors:** Edwin A. Atley

**Affiliations:** Cincinnati


### Original.

1. *Patience and Experience*, versus *Temerity and Ignorance.*—Early on the morning of the 22d, I was roused from bed by a gentleman, who begged me instantly to accompany him, with my *instruments*, for that his wife was in labour, dangerously ill, and the practitioner in attendance was desirous of my immediate assistance.

I accordingly hastened to *arm* myself, and go to the relief of the sufferer. On my way thither, I learned that one of the eminent *Steam* practitioners was the *Accoucheur*, who had been in attendance at intervals for two days, and could do no more.

On entering the apartment of the patient, I found her surrounded by six or seven of her female friends, all of whom were in distress and perturbation, anticipating consequences which neither the appearance nor actual state of the woman warranted. I inquired of the gentleman in attendance, what was the state of the labour, and what the presentation ? He answered that the head of the child was low down, and the presentation *natural, the face downwards.* On examination, I found the os tincæ dilated to almost the diameter of a dollar, rather rigid, the membranes not as yet ruptured, very little tense ; and through them perceived the vertex presenting above the superior strait, apparently in the first position of Baudeloque.

Under these circumstances, it is plain, that there was neither necessity for, nor possibility of, the application of forceps ; and as the strength of the patient, notwithstanding some medicaments to *keep up her heat*, was not much exhausted, I advised quiet and *patience*, assuring the distressed husband, that the labour was likely to terminate in a few hours favorably both to the mother and child.

My professional engagements requiring me to leave the interesting sufferer, I went away, after promising to see her again as soon as possible. But in my absence, which was probably an hour, great alarm was again excited ; the husband was sent, post haste, to my dwelling, after me, but not finding me at home, ran to two or three places before he found a physician. I had however returned before the physician arrived, who was my respected friend Dr. RIVES, whom the poor distressed husband had urged to give help immediately, for his wife was dying.

An explanation of matters soon induced Dr. Rives to withdraw, which indeed his business made him desirous to do, and on inquiry, I found all parties needlessly alarmed. Prescribing another dose of patience, I seated myself by her, discovered the os tincæ much more relaxed, and two or three strong pains ruptured the membranes. After witnessing three or four powerful throes, to make little impression on the head of the child, I *ventured* to propose *venæsection* ! which, after some demur on the part of the patient, was consented to. I accordingly detracted about 16 ounces of blood from the arm ; in consequence of which the pains operated efficiently and in about an hour afterwards I delivered her—neither by *forceps*, nor *crotchet* nor *fillet*—of a fine boy, and saw the grateful patient comfortably placed in bed.

On visiting her, next day, I found the mother and infant well.

EDWIN A. ATLEY.

Cincinnati, June, 1830.

